# Lipophilic Fe(III)-Complex with Potent Broad-Spectrum Anticancer Activity and Ability to Overcome Pt Resistance in A2780cis Cancer Cells

**DOI:** 10.3390/molecules28134917

**Published:** 2023-06-22

**Authors:** Nalin Abeydeera, Morgan Stilgenbauer, Bishnu D. Pant, Khalil Mudarmah, Thiloka M. Dassanayake, Yao-Rong Zheng, Songping D. Huang

**Affiliations:** 1 Department of Chemistry and Biochemistry, Kent State University, Kent, OH 44240, USA; nkekiriw@kent.edu (N.A.); bpant@kent.edu (B.D.P.); kmudarma@kent.edu (K.M.); tdassana@kent.edu (T.M.D.); 2 Department of Chemistry, Jazan University, Jazan 45142, Saudi Arabia

**Keywords:** Iron, Fe(III) coordination compound, Fenton catalyst, ROS signaling pathways

## Abstract

Although iron is essential for all forms of life, it is also potentially toxic to cells as the increased and unregulated iron uptake can catalyze the Fenton reaction to produce reactive oxygen species (ROS), leading to lipid peroxidation of membranes, oxidation of proteins, cleavage of DNA and even activation of apoptotic cell death pathways. We demonstrate that Fe(hinok)_3_ (hinok = 2-hydroxy-4-isopropyl-2,4,6-cycloheptatrien-1-one), a neutral Fe(III) complex with high lipophilicity is capable of bypassing the regulation of iron trafficking to disrupt cellular iron homeostasis; thus, harnessing remarkable anticancer activity against a panel of five different cell lines, including Pt-sensitive ovarian cancer cells (A2780; IC_50_ = 2.05 ± 0.90 μM or 1.20 μg/mL), Pt-resistant ovarian cancer cells (A2780cis; IC_50_ = 0.92 ± 0.73 μM or 0.50 μg/mL), ovarian cancer cells (SKOV-3; IC_50_ = 1.23 ± 0.01 μM or 0.67 μg/mL), breast cancer cells (MDA-MB-231; IC_50_ = 3.83 ± 0.12 μM or 2.0 μg/mL) and lung cancer cells (A549; IC_50_ = 1.50 ± 0.32 μM or 0.82 μg/mL). Of great significance is that Fe(hinok)_3_ exhibits unusual selectivity toward the normal HEK293 cells and the ability to overcome the Pt resistance in the Pt-resistant mutant ovarian cancer cells of A2780cis.

## 1. Introduction

The successful introduction of cisplatin and its several derivatives to the clinical treatment of various solid tumors has almost made anticancer metallodrugs synonymous with Pt coordination compounds [1,2,3,4,5]. Despite the severe toxic side effects, including poor selectivity toward normal cells and the emergence of Pt resistance, the prominence of such DNA-targeting Pt drugs in cancer chemotherapy remains unchallenged [6,7]. On the other hand, the discovery and development of non-Pt anticancer metallodrugs with alternative mechanisms of action to lower the side effects and/or to improve selectivity toward normal cells, as well as to overcome Pt resistance, have been the perennial goals of research in bioinorganic chemistry [8,9,10,11]. Of particular note are a large number of iron complexes that have been intensively investigated for anticancer applications after the in vitro anticancer activity of ferrocenium picrate and ferrocenium trichloroacetate was first discovered in 1984 by Kopf-Maier and co-workers [12]. Thus far, over 200 research articles reporting the anticancer properties of various iron complexes with diverse ligands have appeared in the literature [13,14]. However, no existing iron compounds have advanced to the stage of human clinical studies for cancer treatment, due largely to the lack of sufficient selectivity and safety profile. As iron is known as a double-edged sword in biology, targeting the vulnerability of cellular iron uptake and transport may prove to be highly desirable for the benefit of the discovery and development of iron-based anticancer drugs. Specifically, iron is required by almost all forms of life as an essential micronutrient in vital physiological processes including cellular replication, metabolism and growth, while it is potentially toxic to cells due to its catalytic activity in the Fenton reaction to produce reactive oxygen species (ROS) [15,16,17]. The deleterious cellular effects of ROS on crucial cellular components include lipid peroxidation of membranes, oxidation of proteins, cleavage of DNA and even activation of apoptotic cell death pathways referred to as ferroptosis. Consequently, evolution has rendered biological cells sophisticated cellular machinery to tightly regulate iron uptake, transport and storage [15]. In vertebrates, the tight regulation of iron uptake and transport at the cellular level is achieved through transferrin receptor-mediated endocytosis [18,19]. First, the extracellular Fe(III) is bound to apo-transferrin the iron-transporter protein with high affinity for Fe(III) than for Fe(II) for protection of Fe(III) against trans-metalation and ROS production. The protective coordination environment of transferrin is imparted by the existence of two octahedral binding pockets per each protein molecule formed by two sets of six hard Lewis base donor atoms, i.e., 1 N- and 5 O-donor atoms. Second, the cellular uptake of Fe(III)–transferrin complex occurs via the transferrin receptor-mediated endocytosis, a crucial act by mammalian cells to maintain cellular iron homeostasis [20]. Third, the intracellular release of iron from the Fe(III)–transferrin complex is achieved by the consecutive action of a proton pump to lower the pH in order to protonate the donor atoms and of the metal center reduction by ferric reductase and/or antioxidants [20,21,22]. A careful examination of such pathways of iron uptake and transport in mammalian cells reveals a vulnerability that can be targeted by a biomimetic strategy. Hence, we hypothesize that if a lipophilic Fe(III)–chelator with hard Lewis base donor atoms that can readily be protonated (i.e., with a relatively high p*K_a_*) is used, a large difference in ligand affinity for extracellular Fe(III) and intracellular Fe(II) can be realized, to allow for easy intracellular release of Fe(II) upon simultaneous acidification of the complex and reduction of Fe(III) once iron is transported across the cell membrane in the form of a lipophilic Fe(III)–complex, thus effectively bypassing the regulation of cellular iron homeostasis, which in turn should trigger the production of ROS in cancer cells to cause cell death [15]. It was recently reported that a highly lipophilic tropolone derivative isolated from the heartwood of Taiwanese hinoki and referred to as hinokitiol (2-hydroxy-4-isopropyl-2,4,6-cycloheptatrien-1-one or β-thujaplicin) exhibits a remarkable capability to transport iron across mammalian cells with missing iron-transporter proteins in the form of Fe(hinok)_3_ for potential treatment of iron deficiency [23]. In this publication, we report on our exploration of using this ligand as a stealth iron-carrier to target the vulnerability of iron homeostasis in cancer cells. We show that Fe(hinok)_3_ is capable of bypassing the tight regulation of iron trafficking to disrupt cellular iron homeostasis. As a result, Fe(hinok)_3_ exhibits potent broad-spectrum anticancer activity against five different cell lines. Furthermore, we provide evidence to demonstrate that Fe(hinok)_3_ exhibits lower cytotoxicity in the human embryonic kidney cells (HEK 293) than in cancer cells, indicating that wider therapeutic windows may exist for Fe(hinok)_3_ as an anticancer agent as opposed to cisplatin. We also show that Fe(hinok)_3_ has the ability to overcome Pt resistance in the A2780cis ovarian cancer cells. To the best of our knowledge, Fe(hinok)_3_ is the first example of a metal complex to simultaneously encompass all three desirable characteristics, i.e., higher in vitro cytotoxicity and better selectivity toward normal cells than cisplatin as well as the ability to overcome Pt resistance, three good deeds that have rarely been performed by a single compound thus far.

**Figure 1:**
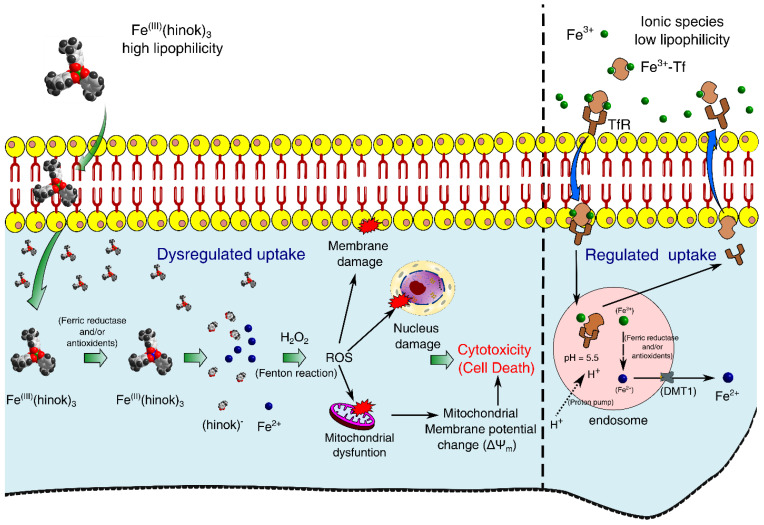
Proposed mechanism of action by Fe(hinok)_3_ against cancer cells via the intracellular ROS production that in turn causes damage to nuclear DNA, mitochondria and cell membrane to trigger apoptotic cell death.

Our experimental results have led us to propose a mechanism of action by Fe(hinok)_3_ in killing different cancer cells where several ROS signaling pathways triggered by the Fenton reaction are primarily responsible for cell death. First, Fe(III) ion in Fe(hinok)_3_ is reduced to Fe(II) by a ferric reductase or/and antioxidants after the entire Fe(hinok)_3_ is transported across the cell membrane [24], which causes Fe(II) to be released from this Fe(III) complex. Second, the coordinatively unsaturated Fe^2+^ ion released from the complex reacts with intracellular H_2_O_2_ to produce the hydroxyl free radical via the Fenton reaction in the form of ROS production. The latter can enter the nucleus to cause ROS-associated damage in DNA; meanwhile, mitochondria appear to be another target of the extrinsic ROS production, resulting in mitochondrial damage including alterations of mitochondrial membrane potential as well as other potential damages, such as damage in NADH dehydrogenase, cytochrome c oxidase, and ATP synthase. At a higher concentration, Fe(hinok)_3_ can also cause cell membrane damage (Figure 1). 

## 2. Results and Discussion

### 2.1. Synthesis and Characterization of Fe(hinok)_3_

We recently published the synthesis, spectroscopic and X-ray powder diffraction characterization of Fe(hinok)_3_ along with the studies of the antimicrobial activity of this Fe(III) complex [25]. As a matter of fact, the remarkable ability of hinokitiol as a stealth ionophore to carry Fe(III) across the bacterial cell membrane prompted us to investigate this natural ligand to perform a similar action in cancer cells, thus harnessing the well-known iron toxicity for anticancer applications. Although the data on the synthesis and characterization of this complex can be found in the above publication, a brief description of its synthesis is given here. Iron (III) chloride (2.03 mmol) and hinokitiol (6.15 mmol) were placed in a 50 mL beaker with a stir bar. After 10 mL ethanol was added, the reaction was vigorously stirred for 3 h to give a cherry-red or purple-colored suspension. The product was collected via filtration and washed with ethanol. The full characterization of the product included elemental analysis, UV-Vis, FT-IR and X-ray powder diffraction as described in the publication [25].

### 2.2. Preferential Complexation of Fe(III) by Hinokitiol and Release of Fe(II) upon Reduction

**Figure 2:**
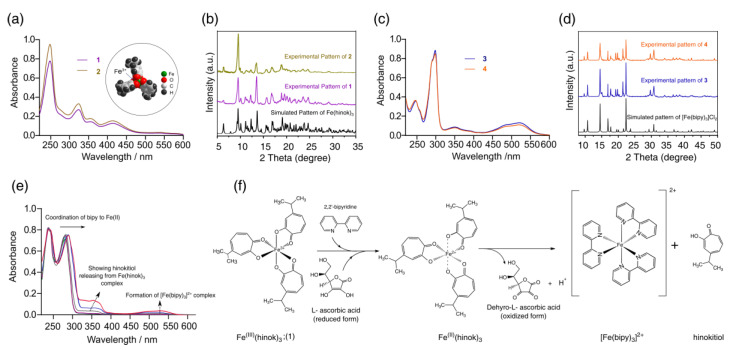
Results of iron complexation and release studies: (**a**) UV−Vis spectra of product **1** and **2** with the molecular structure of Fe(hinok)_3_ depicted by the space-filling model in the insert; (**b**) observed powder XRD patterns of product **1** and **2** in comparison with the simulated powder XRD patterns of Fe(hinok)_3_; (**c**) UV−Vis spectra of product **3** and **4**; (**d**) observed powder XRD patterns of product **3** and **4** in comparison with the simulated powder XRD patterns of [Fe(bipy)_3_]Cl_2_; (**e**) UV−Vis spectra of the reaction between Fe(hinok)_3_ and bipy in the presence of ascorbic acid, showing the formation of [Fe(bipy)_3_]^2+^ upon the reduction of Fe(III) and release of Fe(II) from Fe(hinok)_3_ (spectra were collected from 0 to 20 min); and (**f**) the schematic of Fe(II) release from Fe(hinok)_3_ in the presence of a reducing agent and the subsequent formation of [Fe(bipy)_3_]^2+^.

As a bidentate ligand with two O donors, hinokitiol strongly favors the complexation of Fe(III) (*K_f_* = 5.8 × 10^25^) over Fe(II) (*K_f_* = 5.1 × 10^15^) [23]. As a result, when FeCl_3_ was reacted with hinokitiol, Fe(hinok)_3_ was readily formed as the only product from the reaction (product **1**). The elemental analysis, UV-Vis, FT-IR and powder X-ray diffraction (XRD) studies unequivocally confirmed the isolated cherry-red crystalline product to be Fe(hinok)_3_ with purity > 98% (Table S1). As revealed by its molecular structure determined by X-ray single-crystal analysis [26], Fe(hinok)_3_ has an idealized *D*_3_ symmetry with the Fe^3+^ ion deeply encapsulated in an octahedral coordination cavity created by three highly lipophilic aromatic ligands, which essentially conceals the ionic character of the otherwise highly charged Fe^3+^ center, rendering the complex greaseball-like characteristics (see the insert in Figure 2a). On the other hand, when FeCl_2_ was reacted with hinokitiol in the presence of air, the only complex formed in solution was also Fe(hinok)_3_, as confirmed by the UV-Vis spectrum of the solution (Figure 2a). The powder XRD patterns of the isolated product (product **2**) from this reaction further confirmed the identity of product **2** to be Fe(hinok)_3_ that was completely free of any Fe(II)-containing impurity, e.g., the anionic [Fe(hinok)_3_] ^–^ (Figure 2b and Scheme S1). In contrast, the bidentate ligand 2,2′-bipyridine (bipy) strongly favors the complexation of Fe(II) over Fe(III) [27]. Hence, when FeCl_2_ was reacted with bipy, [Fe(bipy)_3_]^2+^ was readily formed from the reaction (product **3**), while when FeCl_3_ was reacted with 3 molar equivalents of bipy in the air, [Fe(bipy)_3_]^2+^ instead of [Fe(bipy)_3_]^3+^ was the only complex formed in solution and isolatable as [Fe(bipy)_3_]Cl_2_ as product **4** in pure form (Figure 2c,d and Scheme S2). Therefore, we could simultaneously investigate the competitive complexation of Fe(III) in the presence of both ligands, test the stability of Fe(hinok)_3_ against demetallation in the presence of bipy and monitor the release of Fe(II) from Fe(hinok)_3_ when the metal center was reduced using solution UV-Vis spectroscopic measurements. Specifically, we mixed FeCl_3_ with 3 molar equivalents of hinokitiol and 3 molar equivalents of bipy in ethanol and monitored the complexation using the UV-Vis spectroscopic technique. The results showed that Fe(hinok)_3_ was the only complex formed in the solution, indicating that Fe(III) has a higher affinity for hinokitiol than bipy (Figure S1). To investigate (i) whether the demetallation could occur in the formed Fe(hinok)_3_ when this Fe(III) complex was challenged by bipy as a mimic of biological ligands; and (ii) whether the release of Fe(II) could occur from Fe(hinok)_3_ under the acidic and reductive conditions similar to those inside the cancer cell using the formation of Fe(II)–bipy as a visual and spectroscopic probe, we reacted Fe(hinok)_3_ with 3 molar equivalents of bipy in the absence and presence of ascorbic acid. The results showed that demetallation could not occur in the presence of bipy alone, but the release of Fe(II) from Fe(hinok)_3_ was instantaneous when ascorbic acid was added to the reaction as an acidifying and reducing agent (Figure 2e).

### 2.3. Cytotoxicity in Different Cancer Cells

**Figure 3:**
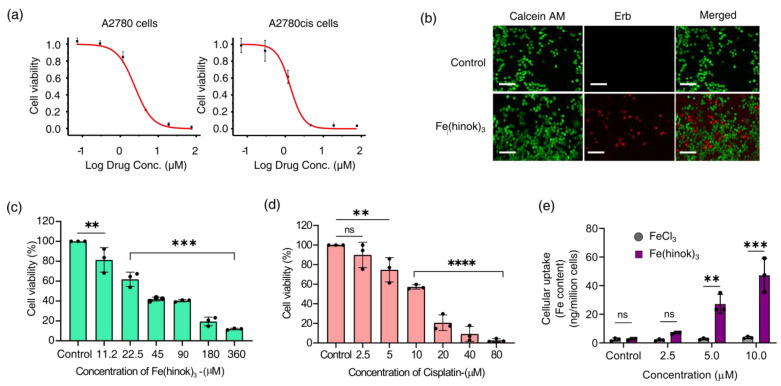
Results of cytotoxicity studies: (**a**) representative killing curves of Fe(hinok)_3_ against A2780 (cisplatin−sensitive) and A2780cis (cisplatin−resistant) ovarian cancer cell lines; (**b**) representative images of live/dead cell assays of A2780cis cells treated with Fe(hinok)_3_ (scale bar = 200 µm); (**c**) cell viability curve of Fe(hinok)_3_ against HEK 293 cells; (**d**) cell viability curve of cisplatin against HEK 293 cells; and (**e**) cellular uptake of Fe(hinok)_3_ in A2780cis as determined by the Fe content in the cell lysate (data presented as mean ± s.d, *n* =3 replicates; ** p < 0.01, *** p < 0.001, **** p < 0.0001 and ns = not significant).

The in vitro anticancer activity of Fe(hinok)_3_ against a panel of five human cancer cell lines was evaluated using the MTT assays. The cell lines employed in this study include A2780 (Pt-sensitive ovarian cancer cells), A2780cis (Pt-resistant ovarian cancer cells), SKOV-3 (ovarian cancer cells), MDA-MB-231 (breast cancer cells) and A549 (lung cancer cells). Cells were treated with Fe(hinok)_3_ for 72 h before their viability was evaluated. The IC_50_ value, meaning the concentration of the drug required to inhibit the growth of cells by 50%, for each of the five cancer cell lines investigated is listed in Table 1, while the representative killing curves against A2780 and A2780cis are shown in Figure 3a. Additionally, three representative killing curves, one for SKOV-3, another for MDA-MB-231 and the third one for A549 are given in Figure S2. The results showed that for the A2780 cell line, the IC_50_ value of Fe(hinok)_3_ is slightly higher than that of cisplatin, while for each of the remaining four cell lines, Fe(hinok)_3_ exhibits a lower IC_50_ value than the counterpart of cisplatin [28]. It is worth noting that in the A2780cis cell line, the IC_50_ of Fe(hinok)_3_ (0.92 ± 0.73 μM or 0.50 μg/mL) is approximately 14 times lower than that of cisplatin (13.19 ± 1.84 μM); in the SKOV-3 cell line, the IC_50_ of Fe(hinok)_3_ (1.23 ± 0.01 μM or 0.67 μg/mL) is also approximately 14 times lower than that of cisplatin (16.31 ± 3.92 μM); in the MDA-MB-231 cell line, the IC_50_ of Fe(hinok)_3_ (3.83 ± 0.12 μM or 2.0 μg/mL) is more than 6 times lower than that of cisplatin (23.68 ± 1.74 μM) and in the A549 cell line, the IC_50_ of Fe(hinok)_3_ (1.50 ± 0.32 μM or 0.82 μg/mL) is almost 10 times lower than that of cisplatin (12.03 ± 1.33 μM). More importantly, Fe(hinok)_3_ exhibits the ability to overcome the Pt resistance in A2780cis. The A2780 (cisplatin-sensitive) and A2780cis (cisplatin-resistant) are a couple of ovarian cancer cell lines commonly used to evaluate the resistance factor (RF) based on the ratio of IC_50_(A2780cis)/IC_50_(A2780) as an indicator. For comparison, the value of the RF of cisplatin in these two cell lines is 8.20 [28], while the value of RF for Fe(hinok)_3_ is 0.45 < 1, confirming that there is no cross-resistance existing between cisplatin and Fe(hinok)_3_. Intriguingly, these results indicate that the A2780cis cells are even more susceptible to Fe(hinok)_3_ than the A2780 cells. Additionally, the anticancer activity of Fe(hinok)_3_ was studied by a live/dead cell assay using fluorescent microscopy with ethidium homodimer (Erb) and a staining of acetomethoxycalcein, or calcein AM. The live cells treated with calcein AM can result in a green fluorescence signal, whereas the dead cells exhibit a red signal due to the ethidium homodimer. The A2780cis cells treated with Fe(hinok)_3_ at the concentration of 1.25 μM for 24 h showed significant cell death (Figure 3b). Overall, we conclude that Fe(hinok)_3_ is more active than cisplatin against four out of the five cancer cell lines examined and exhibits the usual ability to overcome the Pt resistance in A2780cis ovarian cancer cells.

### 2.4. Cytotoxicity in the Human Embryonic Kidney Cells

To estimate the therapeutic index of Fe(hinok)_3_ in these five cancer cell lines, we evaluated the cytotoxicity of Fe(hinok)_3_ in human embryonic kidney cells (HEK 293) (Figure 3c,d) in comparison with cisplatin. The therapeutic index (T.I. = $\frac{IC_{50}\left(\mathrm{normal}\;\mathrm{cells}\right)}{IC_{50}\;\left(\mathrm{cancer}\;\mathrm{cells}\right)\;}$) values of Fe(hinok)_3_ in comparison with the T.I. values of cisplatin for five cancer cells are calculated and given in Table 1. Specifically, the T.I. is 16.67 for Fe(hinok)_3_ vs. 9.37 for cisplatin in A2780 cells; the T.I. is 40.00 for Fe(hinok)_3_ vs. 1.18 for cisplatin in A2780cis cells; the T.I. is 30.78 for Fe(hinok)_3_ vs. 0.95 for cisplatin in SKOV-3 cells; the T.I. is 10.20 for Fe(hinok)_3_ vs. 0.65 for cisplatin in MDA-MB-231 cells; and the T.I. is 26.67 for Fe(hinok)_3_ vs. 1.30 for cisplatin in A549 cells, suggesting that wider therapeutic windows may exist in these cancer cell lines for Fe(hinok)_3_ than cisplatin as a cancer chemotherapeutic. In a word, the cancer cells of all the above five cell lines are more susceptible to Fe(hinok)_3_ than the normal HEK 293 cells are, indicating that the noncancer cells of HEK 293 are better equipped than cancer cells to counteract the deleterious effect of intracellular ROS production. It is well known that the antioxidant defense mechanisms in normal cells involve the action of both non-enzymatic antioxidants such as glutathione, vitamin A, vitamin C, vitamin E, etc., and antioxidant enzymes such as catalase, superoxide dismutase and glutathione peroxidase for maintaining redox homeostasis [29,30]. In sharp contrast, cancer cells usually possess elevated ROS concentrations due largely to the oncogenic stimulation, increased metabolic activity and mitochondrial malfunctioning in comparison with normal cells. Hence, the threshold of triggering ROS-initiated apoptosis is lower in cancer cells than in normal cells.

### 2.5. Cellular Uptake of Fe(hinok)_3_

To examine the cell membrane penetration of Fe(hinok)_3_, we measured its cellular uptake by quantifying the intracellular Fe contents in the cells that were treated with Fe(hinok)_3_. To ensure that cells remain viable to actively uptake Fe(hinok)_3_ in such experiments, A2780cis cells were seated in the wells at a higher density of 2 × 10^5^ cells/well, as opposed to the density of 2 × 10^4^ cells/well used in the MTT assays and incubated for 6 h with either Fe(hinok)_3_ as the treatment group or FeCl_3_ as the reference group, respectively. The cellular Fe contents were determined using atomic absorption spectrometry (AAS). The results showed that at the concentration of 2.5 µM of Fe(hinok)_3_ or FeCl_3_, the intracellular level of Fe in the treatment group was more than tripled (6.97 ± 0.302 ng/million cells) compared to that in the reference group (2.02 ± 0.339 ng/million cells), while at the concentration of 5.0 µM of Fe(hinok)_3_ or FeCl_3_, there was a 25-fold increase in the intracellular level of Fe, i.e., 27.21 ± 3.36 ng/million cells in the treatment group vs. 2.42 ± 0.550 ng/million cells in the reference group, and at the concentration of 5.0 µM of Fe(hinok)_3_ or FeCl_3_, a 45-fold increase in the intracellular level of Fe was observed, i.e., 47.32 ± 5.91 ng/million cells in the treatment group vs. 2.96 ± 0.394 ng/million cells in the reference group. It should be noted that the cells treated with FeCl_3_ at the above three ascending molar concentrations showed no statically significant increase in the intracellular Fe level (Figure 3e). We attribute such remarkable uptake of iron in the treatment group to the electrically neutral and highly lipophilic nature of Fe(hinok)_3_. Although hinokitiol itself is a slightly polar molecule (dipole moment = 4.04 D), upon forming a *D*_3_ octahedral complex with Fe(III), the molecular dipole moment of the complex vanishes, rendering the complex greaseball-like characteristics with Log*p* = 1.71 [23]. Hence, the observed facile internalization of Fe(hinok)_3_ in comparison with FeCl_3_ is consistent with the high lipophilicity of this Fe(III) complex.

### 2.6. The Effect of Fe(hinok)_3_ on the Mitochondrial Membrane Potential (MMP) in A2780cis Cells

**Figure 4:**
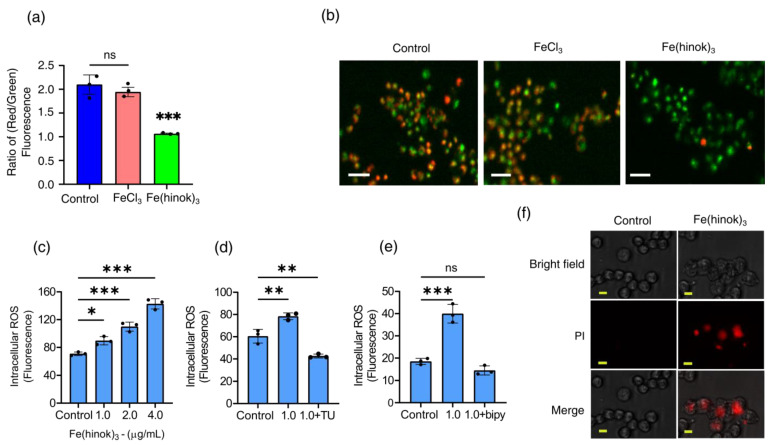
Assessment of mitochondrial depolarization using JC–1 dye: (**a**) the change in the ratio of red/green fluorescence signals in A2780cis cells treated with FeCl_3_ and Fe(hinok)_3_ each at the concentration of 1.0 µM over 24 h; (**b**) representative microscopic images of JC–1 fluorescent signals in A2780cis cells treated with FeCl_3_ and Fe(hinok)_3_ each at 1.0 μM, (scale bar = 100 µm); (**c**) production of ROS following Fe(hinok)_3_ treatment with varying concentrations in A2780cis cells; (**d**) effect of free radical scavenger thiourea (TU) on cell viability; and (**e**) effect of Fe(II) chelator (bipy) on the intracellular ROS production in A2780cis cells (data presented mean ± s.d, *n* = 3 replicates; * *p* < 0.05, ** p < 0.01, *** p < 0.001 and ns = not significant); and (**f**) effect of Fe(hinok)_3_ on the cell membrane permeability and damage in the A2780cis cells showing representative microscopic images of PI in the A2780cis cells treated with Fe(hinok)_3_ at 8 μM. Differential interference contrasts microscopic (DIC) images (top), fluorescence images (middle) and merged images of the A2780cis cells (bottom) (scale bar = 30 µm).

Since a number of structural and functional changes of mitochondria are often manifested by an alteration in the mitochondrial membrane potential (MMP) [31], we then set out to examine whether Fe(hinok)_3_ could affect mitochondrial membrane function by measuring the change in its membrane potential with FeCl_3_ as a reference control using the fluorescence probe 5,5,6,6′-tetrachloro-1,1′,3,3′ tetraethylbenzimi-dazoylcarbocyanine iodide (JC-1 dye). A healthy mitochondrial membrane is negatively charged, and the potential (ΔΨm) is decreased when damaged by toxic substances. Because JC-1 dye carries a positive charge and can stain mitochondria, fluorescent signals obtained from the JC-1 stained cells can be used to determine the change in the ratio of red/green fluorescence to probe the mitochondrial membrane integrity [32]. As shown in Figure 4a,b, the ratio of red/green fluorescence signals was reduced in the cells treated with Fe(hinok)_3_, while the cells treated with FeCl_3_ at the corresponding molar concentration showed no statically significant change in the ratio of red/green fluorescence signals. Specifically, A2780cis cells treated with Fe(hinok)_3_ at the concentration of 1.0 μM for 24 h showed a decrease in the red/green fluorescence intensity ratio indicative of depolarization/disruption of the mitochondrial membrane, while cells treated with FeCl_3_ as a reference control resulting increase in the ratio of red/green fluorescence intensity (Figure 4b). These results indicated that Fe(hinok)_3_ can depolarize the mitochondrial membrane and decrease the membrane potential in the A2780cis cells, thus contributing to the cause of cell death. In sum, both Fe content measurements by AAS and fluorescent imaging studies using the JC-1 dye indicate that Fe(hinok)_3_ can accumulate in mitochondria and trigger mitochondrial damage.

### 2.7. Measurements of Intracellular Generation and Quenching of ROS

The anticancer activity of Fe(hinok)_3_ is most likely to stem from its ability to deliver iron to the intracellular free iron store, nucleus and mitochondria, which in turn triggers the Fenton reaction to produce ROS [33]. Therefore, we examined the level of ROS production in A2780cis cells that were treated with varying amounts of Fe(hinok)_3_ and compared with the level of ROS production in the untreated cells as the control using the DCFHDA (2′,7′-dichlorofluorescein diacetate) cellular ROS assays. Our results showed that there was a dose-dependent increase in ROS production in the A2780cis cells treated with Fe(hinok)_3_, suggesting that the cell death is primarily attributable to ROS generation (Figure 4c). This notion was further supported by the cell rescue experiment using thiourea, an effective ROS scavenger known to be protective against ROS attack by reducing the intracellular accumulation of ROS. To adequately demonstrate that Fe(hinok)_3_ mediated ROS production was responsible for the observed cell death in A2780cis cells, we also studied the effects of free radical scavengers and iron chelators on ROS production. Specifically, TU at a predetermined concentration was used to quench the ROS production in our studies [34]. We found that the A2780 cells were protected from the ROS attack in the presence of TU, although the cell death could not be fully prevented, suggesting that the post-generation scavenging of intracellular ROS could not be completely achieved by TU (Figure 4d). We then tested whether the intracellular ROS generation could be completely inhibited in the presence of 2,2′-bipyridine (bipy), a highly effective Fe(II) chelator that is known to passivate the Fenton catalytic activity of free iron. The results showed that the intracellular ROS generation could be completely quenched to allow for a full restoration of cell viability (Figure 4e), confirming that Fe(II) is responsible for intracellular ROS production.

### 2.8. The Effect of Fe(hinok)_3_ on the Membrane Integrity of A2780cis Cells

We next examined whether Fe(hinok)_3_ can directly damage the cell membrane. This was assessed by quantifying the uptake of propidium iodide (PI), a membrane-impermeable dye that is known to be unable to enter an intact cell unless the cell membrane is compromised and produces fluorescence signals upon binding to nucleic acids. As shown in Figure 4f, the A2780cis cells at the density of 2 × 10^5^ cells/well were treated with FeCl_3_ or Fe(hinok)_3_ each at concentrations of 1.0 µM for 24 h. The results showed that the A2780cis cells treated with Fe(hinok)_3_ exhibited significantly increased uptake of the PI dye when compared with the cells treated with FeCl_3_ and the untreated cells in the control group. Furthermore, the bright-field images clearly showed cell membrane damage in the A2780cis cells of the treatment group when compared with those of the control group, suggesting there is a membrane-lytic property of Fe(hinok)_3_ due to its ability to generate ROS that causes lipid peroxidation of the membrane.

### 2.9. Apoptosis Assay

**Figure 5:**
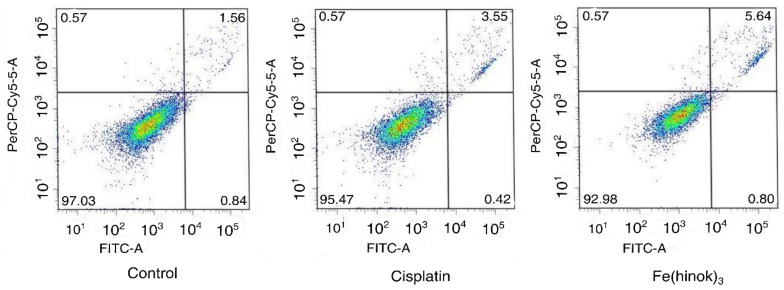
Flow cytometric analysis of apoptotic events in A2780cis cells treated with cisplatin (10.0 µM) and Fe(hinok)_3_ (1.0 μM) for 72 h.

Finally, to determine whether Fe(hinok)_3_ could induce apoptosis, we investigated apoptotic events in A2780cis cells treated with Fe(hinok)_3_ using a FITC-annexin V/PI co-staining flow cytometric assay. Briefly, cells were co-stained with FITC-annexin V and PI after they were treated with Fe(hinok)_3_ and the cellular response was measured with a flow cytometer. FITC-annexin V, a green fluorescence dye-conjugated antibody, is used for the flow cytometric analysis of apoptosis due to its ability to selectively recognize the externalized phosphatidylserine (PS) that occurred at the early of apoptosis. On the other hand, the PI dye, a fluorescence flow cytometric viability probe, is used to distinguish viable from nonviable cells, i.e., viable cells with intact membranes can exclude PI, while dead cells with permeable membranes can stain positive. As shown in Figure 5, Fe(hinok)_3_ specifically, after incubating the cancer cells with 1 µM of Fe(hinok)_3_ for 72 h, about 5.64% of cells were in the late-stage apoptosis. In comparison, it requires approximately 10 times the concentration for cisplatin to achieve a comparable effect, ca. 3.55% cells in the late-stage apoptosis. These results are consistent with the findings that A2780cis cells are resistant to cisplatin, while Fe(hinok)_3_ can readily overcome such resistance developed in these ovarian cancer cells.

## 3. Conclusions

In summary, we have demonstrated that Fe(hinok)_3_ exhibits broad-spectrum in vitro anticancer activity that is higher or at least comparable to cisplatin against a panel of five human cancer cell lines. Furthermore, we have shown that the origin of such potent in vitro anticancer activity of Fe(hinok)_3_ is associated with ROS damage to mitochondria and the cell membrane. Because the cellular targets and the mode of action of Fe(hinok)_3_ are drastically different and nonoverlapping with those of cisplatin, Fe(hinok)_3_ has the distinct ability to overcome Pt resistance in the A2780cis ovarian cancer cells. We have also proven that Fe(hinok)_3_ shows unusual selectivity toward the normal HEK293 cells because cancer cells usually possess elevated ROS concentrations due to the oncogenic stimulation, increased metabolic activity and mitochondrial malfunctioning in comparison with normal cells, rendering the threshold of triggering ROS-initiated apoptosis lower in cancer cells than in normal cells. All these results raise the possibility of harnessing this type of ROS signaling pathways to develop iron-based metallodrugs for cancer chemotherapy.

## 4. Materials and Methods

Material: All chemical reagents were obtained from commercial sources and used without any further purification. FeCl_3_, FeCl_2_.4H_2_O, ethanol, methanol, hinokitiol (ß-thujaplicin/2-Hydroxy-6-propan-2-ylcyclohepta-2,4,6-trien-1-one), nitric acid (HNO_3_), hydrochloric acid (HCl), dimethyl sulfoxide (DMSO), 2,2′ bipyridine (bipy), thiourea (TU) and ascorbic acid were purchased from Sigma-Aldrich. All reactions were carried out under normal atmospheric conditions. Atomic absorption spectroscopy (AAS) measurements were taken on a Buck scientific spectrometer. Fluorescence images were acquired by using inverted microscopy (Olympus IX81, Tokyo, Japan) with a digital camera. Flow cytometry was carried out on a FACSAria™II flow cytometer (BD Biosciences, Franklin Lakes, NJ, USA).

A2780 and A2780cis cell lines were purchased from Sigma-Aldrich and cultured in RPMI 1640 with L-glutamine (Corning), Glendale, AZ, USA) supplemented with 10% FBS (Atlanta Biologicals, Flowery Branch, GA, USA) and 1% Penicillin Streptomycin (Corning). SKOV-3, MDA-MB-231 and A549 cell lines were obtained via American Type Culture Collection, and cultured in DMEM 1 g/L glucose, with L-glutamine and sodium pyruvate (Corning) supplemented with 10% FBS and 1% Penicillin Streptomycin (Corning). All cell lines were cultured at 37 °C under an atmosphere containing 5% CO_2_. Cells were passaged upon reaching 75–85% confluence by trypsinization and split in a 1:5 ratio. All cells were passed every 3 to 4 days and restarted from a frozen stock upon reaching pass number 20.

### 4.1. Reaction between FeCl_2_ and Hinokitiol

Iron (II) chloride tetrahydrate (0.1 mmol) and hinokitiol (0.3 mmol) were stirred in a 50 mL beaker in 10 mL of ethanol for 3 h in the air to give a cherry red, purple colored suspension. The product was collected via filtration and washed with ethanol and dried in a vacuum oven overnight. The collected product, referred to as **2**, was identified by UV-Vis and X-ray powder diffraction techniques as pure Fe(hinok)_3_.

### 4.2. Reaction between FeCl_3_ and 2,2′-Bipyridine

Iron (III) chloride (0.1 mmol) and 2,2′-bipyridine (0.3 mmol) were added to a 50 mL beaker with a magnetic stir bar. After 10 mL ethanol was added, the reaction was vigorously stirred for 3 h to give a red precipitate. The product was collected via a rotary evaporator and washed with diethyl ether three times. The obtained product, referred to as **3**, was identified by UV-Vis and X-ray powder diffraction techniques as pure Fe(bipy)_3_^2+^.

### 4.3. Reaction between FeCl_2_ and 2,2′-Bipyridine

Iron (II) chloride tetrahydrate (0.1 mmol) and 2,2′-bipyridine (0.3 mmol) were added to a 50 mL beaker with a stir bar in the presence of 10 mL ethanol. The reaction mixture was vigorously stirred for 3 h to give a red precipitate. The product was collected via a rotary evaporator and washed with diethyl ether three times. The obtained product, referred to as **4**, was identified by UV-Vis and X-ray powder diffractions as pure Fe(bipy)_3_^2+^.

### 4.4. Reaction between FeCl_3_, Hinokitiol and 2,2′-Bipyridine

Iron (III) chloride (0.1 mmol), hinokitiol (0.3 mmol) and 2,2′-bipyridine (0.3 mmol) were placed in a 50 mL beaker with a stir bar. After 20 mL ethanol was added, the reaction was vigorously stirred for 3 h to give a black precipitate. The product was collected via filtration and washed with ethanol. The product, referred to as **5**, was identified by UV-Vis spectroscopy as pure Fe(hinok)_3_.

### 4.5. Reaction between Fe(hinok)_3_ and 2,2′-Bipyridine in the Presence of Ascorbic Acid

The as-synthesized product of Fe(hinok)_3_ (0.1 mmol) and 2,2′ bipyridine (0.3 mmol) was placed in a 50 mL beaker with 10 mL of ethanol and stirred for 1 h. The reaction progress was monitored using UV-Vis to confirm that there was no reaction between Fe(hinok)_3_ and 2,2′-bipyridine. Thereafter, ascorbic acid (0.1 mmol) was added to the same reaction mixture and vigorously stirred for another 3 h. The product was characterized by UV-Vis as pure Fe(bipy)_3_^2+^.

### 4.6. Cell Viability (MTT) Assays

Cell viability was determined using 3-(4,5-dimethylthiazol-2-yl)-2,5-diphenyltetrazolium bromide (MTT) assays. Cells were seeded in 96-well microplates in 100 μL cell suspensions (2 × 10^4^ cells/well) and incubated for 24 h at 37 °C and 5% CO_2_. Cells in each well were then treated with 100 μL of fresh medium containing varying concentrations of Fe(hinok)_3_ and incubated for 72 h. After the cells were incubated with 10 μL of MTT reagent for 2 h at 37 °C, 100 μL of detergent was added to all wells and the plate was left with cover in the dark for 2 h at 37 °C. The absorbance was measured at 570 nm using a microplate reader (SpectraMax M4). All experiments were performed in triplicate. Finally, the data were analyzed using Origin software and GraphPad Prism software to determine IC_50_ values and the results were represented as percentage of viable cells with respect to the untreated control cells All experiments were performed in triplicate.

### 4.7. LIVE/DEAD Cell Viability Assays

The in vitro efficacy of Fe(hinok)_3_ was examined using the live/dead cell viability assay (molecular probes) in A2780cis ovarian cancer cells. The live/dead cell viability assay kit from molecular probes was used for this assay. A2780cis cells were cultured on 35 mm sterile glass bottom culture dishes for 24 h at 37 °C and grown in RPMI medium supplemented with 10% FBS and 1% penicillin/streptomycin. The cells were then treated with 1.25 µM of Fe(hinok)_3_ for 24 h at 37 °C. First, cells were washed with 1 mL PBS and 1 mL of RPMI (without dye) to remove serum esterase activity. A 5 µL aliquot of calcein AM (4 mM in anhydrous DMSO) and 10 µL ethidium homodimer-1 (2 mM in DMSO/water, 1:4 *v*/*v*) were added to 10 mL of RPMI medium to produce a live/dead working solution. A 2-mL aliquot of live/dead working solution was carefully added to the Petri dishes, which were then incubated at room temperature for 30 min. Finally, the medium of samples was replaced with 1 mL dye-free RPMI before the examination by fluorescence microscopy.

### 4.8. Cellular Uptake

A2780cis cells were seeded in a 6-well plate at a cell density of 2 × 10^5^ cells/well and incubated at 37 °C overnight. The cells were treated with FeCl_3_ or Fe(hinok)_3_ with varying concentrations for 6 h at 37 °C. Cells were washed with 1 mL of phosphate-buffered saline (PBS 1X) three times. The remaining alive cells were harvested by trypsinization and counted. Cells were then digested in 200 µL 75% HNO_3_ at room temperature overnight to destroy the organics and iron ions in this solution were then converted to iron oxide by calcination of the sample at 620 °C for 5 h. The iron oxide obtained was dissolved in aqua regia and the Fe concentrations were analyzed using AAS. All experiments were performed in triplicate.

### 4.9. Determination of the Change in the Mitochondrial Membrane Potential

The change of mitochondrial membrane potential was assayed using fluorescence microscopy and spectrophotometric analysis. Briefly, A2780cis cells were seeded at a density of 2 × 10^4^ cells/well in 35 mm sterile glass bottom culture dishes and incubated overnight. Cells were then treated with Fe(hinok)_3_ or FeCl_3_ as vehicle control (all at 1.0 µM). After 24 h, cells were washed with PBS 1X (pH 7.4) and incubated with a medium containing the JC-1 dye (final concentration 2 µM) for 20 min at 37 °C. Finally, the medium of the samples was replaced with 500 µL dye-free RPMI before the examination by fluorescence microscopy (Olympus IX83). For the spectrophotometric analysis, A2780cis cells were seeded at a density of 2 × 10^4^ cells/well in 96-well plates and incubated overnight. Cells were then treated with Fe(hinok)_3_ or FeCl_3_ as vehicle control (all at 1.0 µM). After 24 h, cells were washed with PBS 1X (pH 7.4) and incubated with a medium containing JC-1 dye (final concentration 2 µM) for 20 min at 37 °C. Finally, the cells were washed and resuspended in 100 µL PBS 1X (pH 7.4) for fluorescent measurements (red 550/600 and green 485/535) using a microplate reader (SpectraMax M4, Molecular Devices LLC, San Jose, CA, USA).

### 4.10. Cell Membrane Permeabilization Assay

The cell membrane permeabilization assay was performed by fluorescence microscopy. A2780cis cells were seeded at a density of 2 × 10^4^ cells/well in 35 mm sterile glass bottom culture dishes and incubated overnight. Cells were then treated with Fe(hinok)_3_ or FeCl_3_ as vehicle control (all at 8 µM). After 24 h, cells were washed with PBS 1X (pH 7.4) and incubated with a medium containing the PI dye (final concentration 2 µM) for 20 min at 37 °C. Finally, the medium of the samples was replaced with 500 µL dye-free RPMI before the examination by fluorescence microscopy (Olympus IX83, Tokyo, Japan).

### 4.11. Measurement of the Levels of Intracellular Total ROS

A2780cis cells were plated in black clear-bottomed 96-well plates at a cell density of 2 × 10^4^ cells/well to assess the production of intracellular ROS levels. The cells were treated with Fe(hinok)_3_ or FeCl_3_ at varying concentrations for 24 h. Afterward, 2′,7′-dichlorofluorescein diacetate (DCFH-DA) dye was added to the medium at a final concentration of 10 µM and incubated in the dark for 30 min. The cells were then washed twice with phosphate buffer solution (PBS 1X; pH 7.4) and resuspended in 100 µL of PBS. The intracellular ROS level was examined by measuring the fluorescence intensity of the solution with a SpectraMax M4. Microplate reader using the 497/529 nm (Excitation/Emission) wavelength. The data were obtained from experiments that were performed independently in triplicate.

### 4.12. ROS Scavenge Assay

A2780cis cells were plated in black clear-bottomed 96-well plates at a cell density of 2 × 10^4^ cells/well to assess the production of intracellular ROS levels. Then, cells were treated with radical scavengers (thiourea-TU-40 mM) or iron chelator (2,2′-bipyridine-bipy −200 μM) and incubated for 30 min to examine the effects of free radical scavengers and iron chelators on ROS production. The cells were treated with Fe(hinok)_3_ and vehicle control for 24 h. Afterward, DCFH-DA dye was added to the medium at a final concentration of 10 µM and incubated in the dark for 30 min. The cells were then washed twice with PBS 1× (pH 7.4) and resuspended in 100 µL of PBS. The intracellular ROS level was examined by measuring the fluorescence intensity of the solution with a SpectraMax M4 Microplate Reader using the 497/529 nm (Excitation/Emission) wavelength. The data were obtained from experiments that were performed independently in triplicate.

### 4.13. Apoptosis Assays

A2780cis cells were seeded in a 6-well plate at a concentration of 2 × 10^5^ cells/well and incubated for 24 h at 37 °C. Then, the cells were treated with Fe(hinok)_3_ (1.0 µM) or cisplatin (10 μM) and untreated control group. Cells were then incubated for 72 h at 37 °C. The medium was collected in clean 15 mL falcon tubes along with washed PBS 1× and pH 7.4 solution. A volume of 1 mL trypsin was added to the wells. After 5 min, cell suspensions were transferred to the falcon tubes that contained the media and PBS 1× and pH 7.4 and centrifuged at 400–500× *g* at 4 °C for 5 min. The cell pellet was re-suspended in 1 mL PBS 1× and pH 7.4 and the cells were counted. The cell pellet was collected again and the appropriate amount of 1× binding buffer was added to make a concentration of 10^6^ cells/mL. Next, 100 μL cell suspensions were added to new 2 mL Eppendorf tubes and 5 μL Annexin V-FITC was added to one tube and 5 μL PI solution was added to another. Cells were gently vortexed and incubated at room temperature for 15 min in the dark; 400 µL 1× binding buffer was added to each Eppendorf tube and the cell suspensions were transferred to flow cytometry tubes. Flow cytometry analysis was done using FL-1 and FL-2 channels on a FACSAria™II flow cytometer (BD Biosciences, Franklin Lakes, NJ, USA).

### 4.14. Statistical Analysis

GraphPad Prism version 8.0 software was used to conduct the statistical analysis. The in vitro bioassays were run with at least three biological replicates. A two-tailed unpaired t-test was used to determine the statistical significance between the two groups. For all analyses, a *p*-value of less than 0.05 was considered to be statistically significant. Data were presented as mean ± standard deviation (mean ± s.d).

## Figures and Tables

**Table 1:** Cytotoxicity profiles of cisplatin and Fe(hinok)_3_ against a panel of human cancer cell lines (72 h, RF = resistance factor) and the therapeutic index (T.I. = IC_50_ (normal cells)/IC_50_ (cancer cells) values of cisplatin and Fe(hinok)_3_ for A2780, A2780cis, SKOV-3, MDA-MB-231 and A549 (data presented mean ± s.d, *n* = 3 replicates).

IC_50_ (µM)/(µg/mL)	A2780	A2780cis	SKOV-3	MDA-MB-231	A549	$\mathrm{Resistant}\;\mathrm{Factor}:\;\frac{IC_{50}\left(A2780cis\right)}{IC_{50}\left(A2780\right)}$
	Ovarian Cancer Sensitive to cisplatin	Ovarian Cancer Resistant to cisplatin	Ovarian Cancer	Breast Cancer	Lung Cancer	
Cisplatin	1.60 ± 0.45 µM	13.19 ± 1.84 µM	16.31 ± 3.92 µM	23.68 ± 1.74 µM	12.03 ± 1.44 µM	8.20
T.I. Cisplatin	9.37	1.18	0.95	0.65	1.30	
Fe(hinok)_3_	2.05 ± 0.90 µM (1.20 µg/mL)	0.92 ± 0.73 µM (0.50 µg/mL)	1.23 ± 0.01 µM (0.67 µg/mL)	3.83 ± 0.12 µM (2.0 µg/mL)	1.50 ± 0.32 µM (0.82 µg/mL)	0.45
T.I. Fe(hinok)_3_	16.67	40.00	30.78	10.20	26.67	

## Data Availability

The data presented in this study are available in the article and its supplementary material.

## Supplementary Materials

The following supporting information can be downloaded at: https://www.mdpi.com/article/10.3390/molecules28134917/s1, Figure S1: UV-Vis spectra of complex formed from the reaction of FeCl_3_ in the presence of hinokitiol (3 molar equivalents) and 2,2′-bipyridine; bipy (3 molar equivalents) in ethanol; Figure S2. Representative killing curves of SKOV-3 (ovarian cancer cells; IC_50_ = 1.23 ± 0.01 μM or 0.67 μg/mL), MDA-MB-231 (breast cancer cells; IC_50_ = 3.83 ± 0.12 μM or 2.0 μg/mL) and A549 (lung cancer cells; IC_50_ = 1.50 ± 0.32 μM or 0.82 μg/mL); Table S1: Elemental analysis results of Fe(hinok)_3_; Scheme S1: Formation of Fe(hinok)_3_ in ethanol from the complexation of Fe(III) or Fe(II) by hinokitiol; Scheme S2. Formation of [Fe(bipy)_3_]^2+^ in ethanol from the complexation of Fe(III) or Fe(II) by 2,2′-bipyridine.
