# Supplementary material for: Lipophilic Fe(III)-Complex with Potent Broad-Spectrum Anticancer Activity and Ability to Overcome Pt Resistance in A2780cis Cancer Cells
Source: Molecules. 2023 Jun 22;28(13):4917. doi: 10.3390/molecules28134917 (PMC10343305; doi:10.3390/molecules28134917)
Supplement: Supplementary file 1 [file molecules-28-04917-s001.zip › molecules-2443672-supplementary.pdf]

Lipophilic Fe(III)-Complex with Potent Broad-spectrum Anticancer Activity and Ability to Overcome Pt Resistance in A2780cis Cancer Cells

Nalin Abeydeera[1], Morgan Stilgenbauer[1], Bishnu D. Pant[1], Khalil Mudarmah[1, 2], Thiloka M. Dassanayake[1], Yao-Rong Zheng[1]* and Songping D. Huang[1]*

[1]Department of Chemistry and Biochemistry, Kent State University, Kent, OH 44240, USA.

[2]Department of Chemistry, Jazan University, Jazan, 45142, Saudi Arabia.

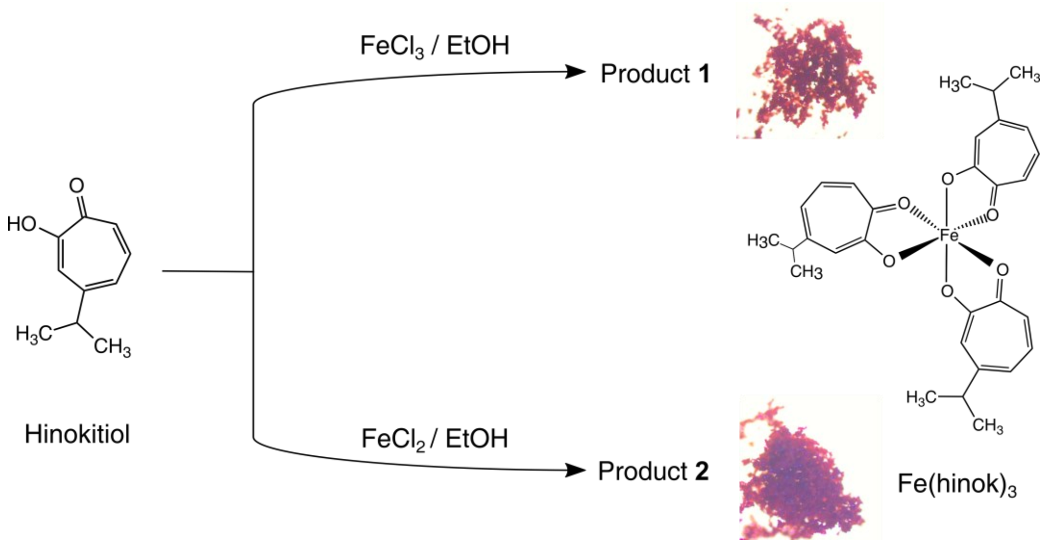

Scheme S1. Formation of Fe(hinok)3 in ethanol from the complexation of Fe(III) or Fe(II) by hinokitiol.

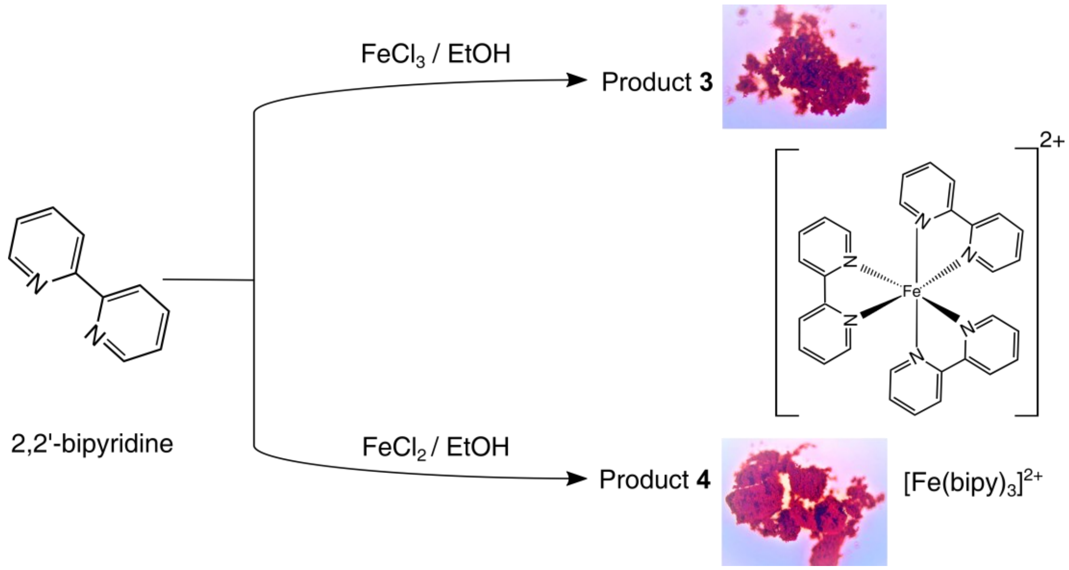

Scheme S2. Formation of $[Fe(bipy)_3]^{2+}$ in ethanol from the complexation of Fe(III) or Fe(II) by 2,2'-bipyridine.

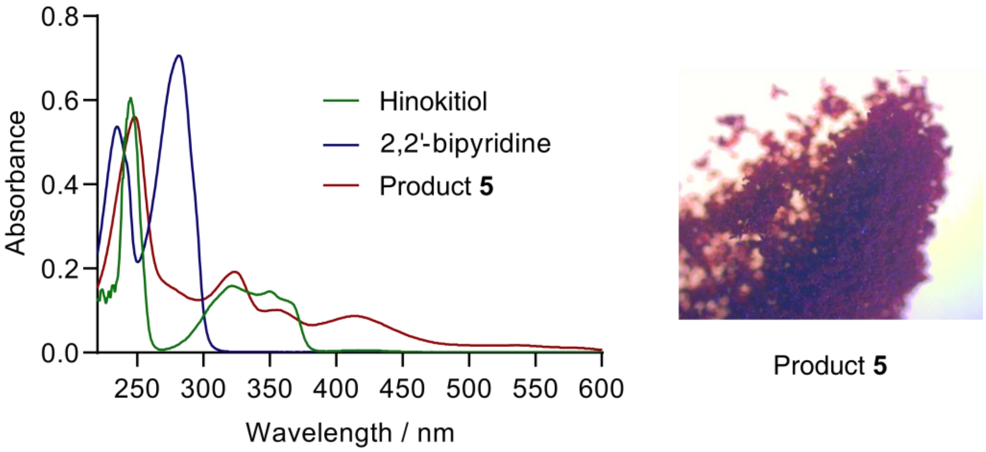

Reaction:

$FeCl_3$ + 3 hinokitiol + 3 bipy ⟶ $Fe(hinok)_3$ **5**

Figure S1. UV-Vis spectra of complex formed from the reaction of $FeCl_3$ in the presence of hinokitiol (3 molar equivalents) and 2,2'-bipyridine; bipy (3 molar equivalents) in ethanol, confirming that the only complex formed is $Fe(hinok)_3$ (left) and a selective photographic image of the isolated product (product **5**) from the reaction (right).

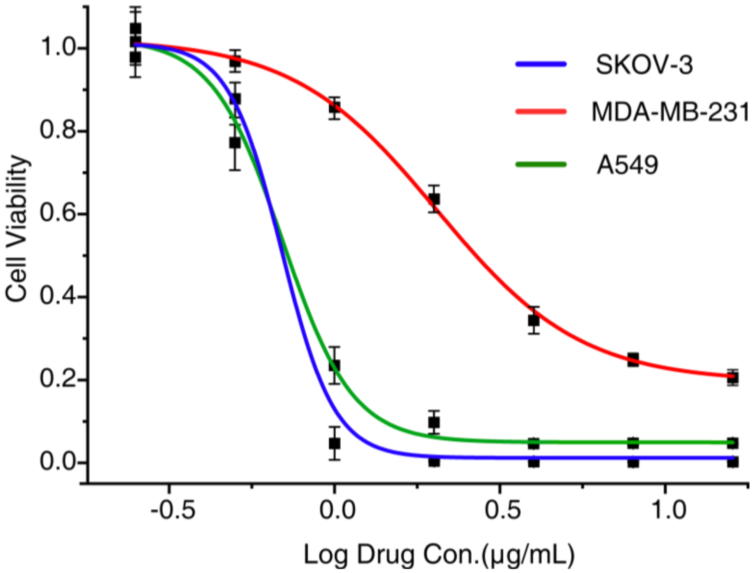

Figure S2. Representative killing curves of SKOV-3 (ovarian cancer cells; IC_{50}=1.23±0.01 μM or 0.67 μg/mL), MDA-MB-231 (breast cancer cells; IC_{50}=3.83±0.12 μM or 2.0 μg/mL) and A549 (lung cancer cells; IC_{50}=1.50±0.32 μM or 0.82 μg/mL).

Table S1. Elemental analysis results of Fe(hinok)$_3$.

Elements	$C_{30}H_{33}O_6Fe$	
	Calculated %	Experimental %
Carbon (C)	66.06	65.84
Hydrogen (H)	6.10	5.96
Iron (Fe)	10.24	10.14
